# Membrane voltage-dependent activation mechanism of the bacterial flagellar protein export apparatus

**DOI:** 10.1073/pnas.2026587118

**Published:** 2021-05-25

**Authors:** Tohru Minamino, Yusuke V. Morimoto, Miki Kinoshita, Keiichi Namba

**Affiliations:** ^a^Graduate School of Frontier Biosciences, Osaka University, Osaka 565-0871, Japan;; ^b^Department of Physics and Information Technology, Faculty of Computer Science and Systems Engineering, Kyushu Institute of Technology, Fukuoka 820-8502, Japan;; ^c^Precursory Research for Embryonic Science and Technology, Japan Science and Technology Agency, Kawaguchi 332-0012, Japan;; ^d^SPring-8 Center, RIKEN, Osaka 565-0871, Japan;; ^e^Center for Biosystems Dynamics Research, RIKEN, Osaka 565-0871, Japan;; ^f^JEOL YOKOGUSHI Research Alliance Laboratories, Osaka University, Osaka 565-0871, Japan

**Keywords:** bacterial flagellum, membrane voltage, proton motive force, type III protein export, *Salmonella*

## Abstract

The proton motive force (PMF) consists of the electric potential difference (Δψ), which is measured as membrane voltage, and the proton concentration difference (ΔpH) across the cytoplasmic membrane. The flagellar protein export machinery is composed of a PMF-driven transmembrane export gate complex and a cytoplasmic ATPase ring complex consisting of FliH, FliI, and FliJ. ATP hydrolysis by the FliI ATPase activates the export gate complex to become an active protein transporter utilizing Δψ to drive proton-coupled protein export. An interaction between FliJ and a transmembrane ion channel protein, FlhA, is a critical step for Δψ-driven protein export. To clarify how Δψ is utilized for flagellar protein export, we analyzed the export properties of the export gate complex in the absence of FliH and FliI. The protein transport activity of the export gate complex was very low at external pH 7.0 but increased significantly with an increase in Δψ by an upward shift of external pH from 7.0 to 8.5. This observation suggests that the export gate complex is equipped with a voltage-gated mechanism. An increase in the cytoplasmic level of FliJ and a gain-of-function mutation in FlhA significantly reduced the Δψ dependency of flagellar protein export by the export gate complex. However, deletion of FliJ decreased Δψ-dependent protein export significantly. We propose that Δψ is required for efficient interaction between FliJ and FlhA to open the FlhA ion channel to conduct protons to drive flagellar protein export in a Δψ-dependent manner.

## Significance

The transmembrane electrical potential difference (Δψ), which is defined as membrane voltage, is used as the energy for many biological activities. For construction of the bacterial flagella on the cell surface, a specialized protein transporter utilizes Δψ to drive proton-coupled protein export, but it remains unknown how. Here, we report that an inactive flagellar protein transporter can be activated by an increase in Δψ above a threshold value through an interaction between FliJ and the transmembrane proton channel protein FlhA. Following activation, the protein transporter conducts protons through the FlhA channel to drive flagellar protein export. This report describes a Δψ-dependent activation mechanism used for a biological function other than voltage-gated ion channels.

The ion motive force (IMF) across the cell membrane is one of the most important sources of biological energy in any cell. The IMF is utilized for many essential biological activities, such as ATP synthesis, solute transport, nutrient uptake, protein secretion, flagella-driven motility, and so on (1). The IMF is the sum of the electrical (Δψ) and chemical (ΔpI) potential differences of ions such as protons (H^+^) (the proton motive force [PMF]) and sodium ions (Na^+^) (the sodium motive force [SMF]) across the membrane and is defined by Eq. **1**:

$$\mathrm{IMF}=V_{m}+\frac{k_{B}T}{q}\;\ln\frac{{\left[\mathrm{ion}\right]}_{\mathrm{in}}}{{\left[\mathrm{ion}\right]}_{\mathrm{ex}}},$$ [1]

where *V*_m_ is Δψ; [ion]_in_ and [ion]_ex_ are the internal and external ion concentrations, respectively; *k*_B_ is Boltzmann’s constant; *T* is the absolute temperature (in kelvins); and *q* is the charge of the ion. The Δψ corresponds to the membrane voltage (2).

The flagellum of the enteric bacterium *Salmonella enterica* serovar Typhimurium (hereafter referred to as *Salmonella*) is a supramolecular motility machine consisting of the basal body, which acts as a bidirectional rotary motor; the hook, which functions as a universal joint; and the filament, which works as a helical propeller. The *Salmonella* flagellar motor is powered by a PMF across the cytoplasmic membrane. The motor consists of a rotor and multiple stator units, each of which acts as a transmembrane proton channel complex. The stator unit converts the proton influx through the channel into the force for high-speed rotation of the long helical filament (3, 4).

For construction of the hook and filament structures at the cell exterior, a specialized protein transporter utilizes the PMF to transport flagellar building blocks to the distal end of the growing flagellar structure. The flagellar protein transporter consists of a PMF-driven export gate complex made of five transmembrane proteins, FlhA, FlhB, FliP, FliQ, and FliR, and an ATPase ring complex consisting of three cytoplasmic proteins, FliH, FliI, and FliJ (*SI Appendix*, Fig. S1) (5, 6). These proteins are evolutionarily related to those of the virulence-associated type III secretion systems of pathogenic bacteria, which inject effector proteins into eukaryotic host cells for invasion (7). Furthermore, the entire structure of the ATPase ring complex is structurally similar to the cytoplasmic F_1_ part of F_O_F_1_-ATP synthase, which utilizes the PMF for ATP synthesis (8–10).

FliI forms a homo-hexamer that hydrolyzes ATP at an interface between neighboring FliI subunits (10–12). FliJ binds to the central pore of the FliI ring (9). ATP hydrolysis by the FliI ATPase not only activates the transmembrane export gate complex through an interaction between FliJ and the C-terminal cytoplasmic domain of FlhA (FlhA_C_) (13, 14) but also opens the entrance gate of the polypeptide channel through an interaction between FliI and the C-terminal cytoplasmic domain of FlhB (FlhB_C_) (15). As a result, the export gate complex becomes an active proton/protein antiporter that couples an inward-directed H^+^ flow with an outward-directed protein export (*SI Appendix*, Fig. S1) (16). When the cytoplasmic ATPase complex becomes nonfunctional, the FlgN chaperone activates the Na^+^-driven export engine of the export gate complex over a wide range of external pH, allowing the export gate complex to drive Na^+^-coupled protein export (17, 18). The transmembrane domain of FlhA (FlhA_TM_) acts as a transmembrane ion channel for the transit of both H^+^ and Na^+^ across the cytoplasmic membrane (17).

A chemical potential gradient of either H^+^ (ΔpH) or Na^+^ (ΔpNa) is required for efficient inward-directed translocation of H^+^ or Na^+^ when FliH and FliI are absent (13, 17). Although the Δψ component is critical for flagellar protein export by the wild-type export gate complex (19), it remains unknown when and how Δψ is used for the flagellar protein export process. To clarify this question, we used the *Salmonella* MMHI0117 [Δ*fliH-fliI flhB(P28T)*] strain (hereafter referred to as ΔHI B*; Table 1) (20), in which the export gate complex uses both Δψ and ΔpNa at different steps of the flagellar protein export process (13, 17). We show that an increase in Δψ generated by an upward shift of the external pH from 7.0 to 8.5 activates flagellar protein export by this mutant even in the absence of ΔpNa, suggesting the presence of a Δψ-dependent activation mechanism for proton-coupled protein secretion by the export gate complex. We also show that an increased Δψ facilitates efficient docking of FliJ to FlhA_C_.

**Table 1.** Summary for flagellar protein export properties of *Salmonella* strains used in this study

Strains	Abbreviated name	External pH	FlgD secretion
SJW1103 (wild type)	WT	7.0	++++
		7.5	+++++
		8.0	+++++
		8.5	+++++
MMHI0117 (ΔfliHI flhB*)	ΔHI B*	7.0	+/−
		7.5	+
		8.0	++++
		8.5	+++++
NH004 (ΔfliHI flhB* Δ*flhA*)	ΔHI B* ΔA	7.0	—
		7.5	—
		8.0	—
		8.5	—
MMHI0017-3 [ΔfliHI flhB* flhA(T490M)]	ΔHI B* A*	7.0	+++++
		7.5	+++++
		8.0	+++++
		8.5	+++++
MMHIJ0117 (ΔfliHIJ flhB*)	ΔHIJ B*	7.0	—
		7.5	—
		8.0	+/−
		8.5	+
MMHIJ0117-3 [ΔfliHIJ flhB* flhA(T490M)]	ΔHIJ B* A*	7.0	+++++
		7.5	+++++
		8.0	+++++
		8.5	+++++

## Results

### Effect of an Increase in Δψ on Flagellar Protein Export by the Transmembrane Export Gate Complex.

**Fig. 1.**
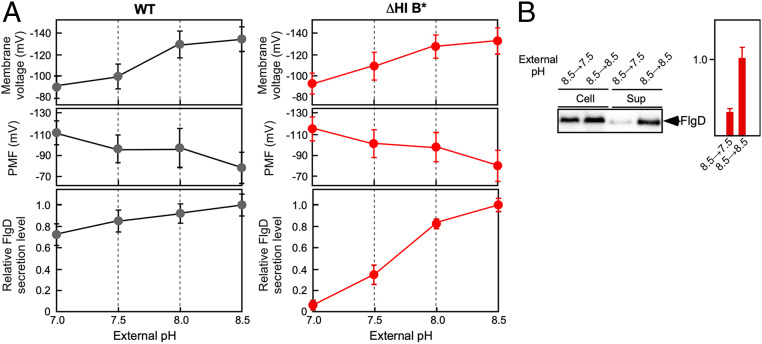
The effect of Δψ on flagellar protein export. (*A*) The relative secretion level of FlgD over an external pH range of 7.0–8.5. The *Salmonella* SJW1103 (wild type, indicated as WT) and MMHI0117 [∆*fliH-fliI flhB(P28T)*, indicated as ∆HI B*] cells were grown at 30 °C in T-broth at external pH values of 7.0, 7.5, 8.0, or 8.5. Whole-cell and culture-supernatant fractions were prepared, followed by SDS-PAGE and immunoblotting with polyclonal anti-FlgD antibody. Relative secretion levels of FlgD were measured. These data are the average from six independent experiments. The membrane potential difference (in millivolts) was measured by using tetramethylrhodamine methyl ester. The vertical bars indicate SDs. The intracellular pH was measured using pHluorin(M153R), and then the total PMF was calculated (*SI Appendix*, Table S1). (*B*) The effect of decreased Δψ on flagellar protein export by the ∆HI B* mutant. The ∆HI B* cells were grown at 30 °C in T-broth (pH 8.5). After washing twice with T-broth (pH 7.5), the cells were resuspended in T-broth at a pH value of 7.5 or 8.5 and incubated at 30 °C for 1 h. The whole-cell (Cell) and culture-supernatant fractions (Sup) were analyzed by immunoblotting with polyclonal anti-FlgD antibody. The relative secretion levels of FlgD were measured. These data are the average from six independent experiments.

The ∆pH and ∆pNa components are required for efficient transit of H^+^ and Na^+^ through the FlhA ion channel, respectively, when FliH and FliI are missing (13, 17). In *Salmonella*, intracellular pH is maintained at about 7.5 over a wide range of external pH (21). Because an external pH higher than 7.5 results in a negative value of ΔpH, bacterial cells autonomously increase Δψ to maintain the total PMF constant as much as possible (22, 23). Consistently, an upward shift of external pH from 7.0 to 8.5 increased Δψ significantly but decreased the total PMF (Fig. 1*A* and *SI Appendix*, Table S1). It has been shown that FliH and FliI make the transmembrane export gate complex robust against a variety of perturbations. Thus, the export gate complex maintains protein transport activity over a wide range of external pH from 6.0 to 8.0 (13, 17).

To clarify the role of Δψ in flagellar protein export, we used the *Salmonella* ΔHI B* strain, in which the B* mutation significantly increases the probability of the substrate entry into the polypeptide channel in the absence of FliH and FliI (20). We chose the hook-capping protein FlgD as a representative export substrate because the level of FlgD secretion by this mutant strain is even higher than the wild-type level (20). The transmembrane export gate complex of the ΔHI B* strain prefers Na^+^ over H^+^ as the coupling ion in an external pH range of 6.0–8.0 (17, 18). Therefore, the ΔHI B* cells were grown in T-broth at external pH values of 7.0, 7.5, 8.0, or 8.5 in the absence of external Na^+^ to examine the Δψ dependence of protein export. The amount of FlgD secreted by the ΔHI B* mutant was analyzed by quantitative immunoblotting with polyclonal anti-FlgD antibody (*SI Appendix*, Fig. S2). The results for all strains are qualitatively summarized in Table 1.

In the Δ*fliH-fliI flhB(P28T)* Δ*flhA* strain (ΔHI B* ΔA; Table 1), the negative control, no FlgD was detected in the culture supernatant (*SI Appendix*, Fig. S2*B*). The relative levels of FlgD secreted by the ΔHI B* cells increased with an increase in Δψ above a threshold value (Fig. 1 *A*, *Right*, and *SI Appendix*, Fig. S2*C*). The full activity of the export gate complex was attained when Δψ reached 1.5-fold above the threshold value.

The Δψ component is essential for flagellar protein export by *Salmonella* wild-type cells (13, 19). Therefore, we tested whether having Δψ above the threshold value also increased the secretion level of FlgD by wild-type cells. The secretion levels of FlgD by wild-type cells were almost constant over an external pH range of 7.0–8.5 (Fig. 1 *A*, *Left*, and *SI Appendix*, Fig. S2*A*), indicating that an increase in Δψ does not facilitate flagellar protein export by the wild-type protein transporter. Thus, the rate of H^+^-coupled protein translocation by the export gate complex is high enough to mask the effect of increased Δψ when FliH and FliI are present.

We found that the amount of FlgD secreted by the ΔHI B* cells increased with an increase in Δψ (Fig. 1 *A*, *Right*). Therefore, we investigated whether a decrease in Δψ by a downward pH shift from 8.5 to 7.0 reversibly decreases the Δψ-dependent flagellar protein transport activity of the ΔHI B* cells. The cells were first grown at pH 8.5, and then the external pH value was shifted from 8.5 to 7.5. This downward pH shift decreased the secretion level of FlgD by about 3.3-fold (Fig. 1 *B*, *Right*), which is consistent with the data shown in Fig. 1 *A*, *Right Lower*. This confirms that the export gate complex becomes a Δψ-dependent export engine when Δψ increases above the threshold.

### Effect of Increased Δψ on the Filament Growth Rate.

**Fig. 2.**
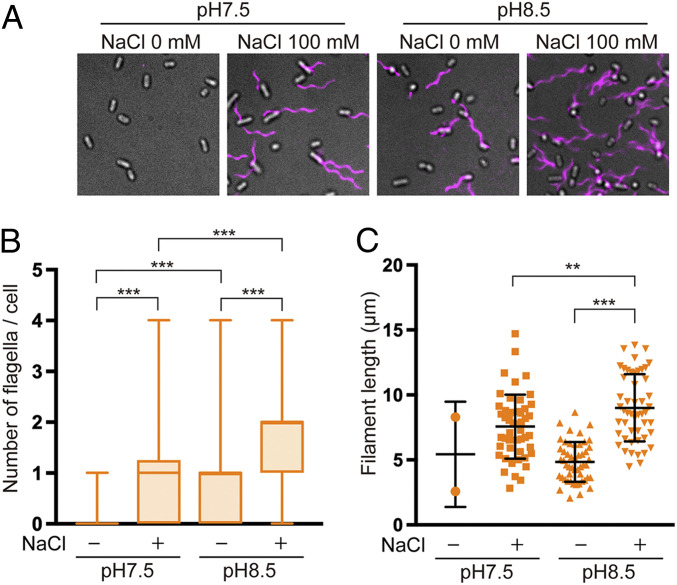
The effect of increased Δψ on flagellar formation. (*A*) Fluorescent images of ∆HI B* cells grown in TB at pH 7.5 or TB at pH 8.5 with or without 100 mM NaCl. Flagellar filaments were labeled with Alexa Fluor 594. The fluorescence images of the filaments labeled with Alexa Fluor 594 (magenta) were merged with the bright-field images of the cell bodies. (*B*) Box plots of the flagellar filaments in the ∆HI B* cells. The lower and upper box boundaries are 25th and 75th percentiles, respectively. The line in the middle of the box shows the median number. The lower and upper error lines indicate the smallest and largest values, respectively. More than 150 cells were counted. (*C*) Scatter plots of the length of the flagellar filaments. The filament length is the average of 50 filaments, and lines represent mean values with SDs. Only two of the ∆HI B* cells had filaments at pH 7.5 in the absence of NaCl. Comparisons between datasets were performed using a two-tailed Student’s *t* test. A value of *P* < 0.05 was considered to be statistically significant. **P < 0.01; ***P < 0.001 (*SI Appendix*, Table S2).

Flagellar building blocks are translocated across the cytoplasmic membrane via the PMF-driven export gate complex, diffuse down the central channel of the growing flagellar structure, and assemble at its distal end (24). Therefore, the flagellar growth rate is determined by the rate of PMF-driven protein translocation by the export gate complex as well as by the diffusion rate of the flagellar building blocks. A decrease in the PMF decreases the rate of filament growth significantly (24). To monitor the Δψ dependence of the export activity in the ΔHI B* mutant, ΔHI B* cells were grown in T-broth at an external pH of 7.5 or 8.5, and their flagellar filaments were labeled with a fluorescent dye, Alexa Fluor 594, to measure the number and lengths of the filaments (Fig. 2 and *SI Appendix*, Table S2). Most of the ΔHI B* cells had no filaments; only 1.0% had a single filament at external pH 7.5 (*n* = 198). In contrast, at an external pH of 8.5, 60.5% of the HI B* cells produced filaments, with an average of 1.3 ± 0.5 per cell (mean ± SD; *n* = 107) (Fig. 2*B*). The average filament length was 4.8 ± 1.5 μm (*n* = 50). These results suggest that the ΔHI B* cells transport flagellar building blocks in a Δψ-dependent manner once their export gate complexes are activated by an increase in Δψ above a threshold value.

### Multicopy Effect of FliJ on Δψ-Dependent Flagellar Protein Export by ΔHI B* Cells.

**Fig. 3.**
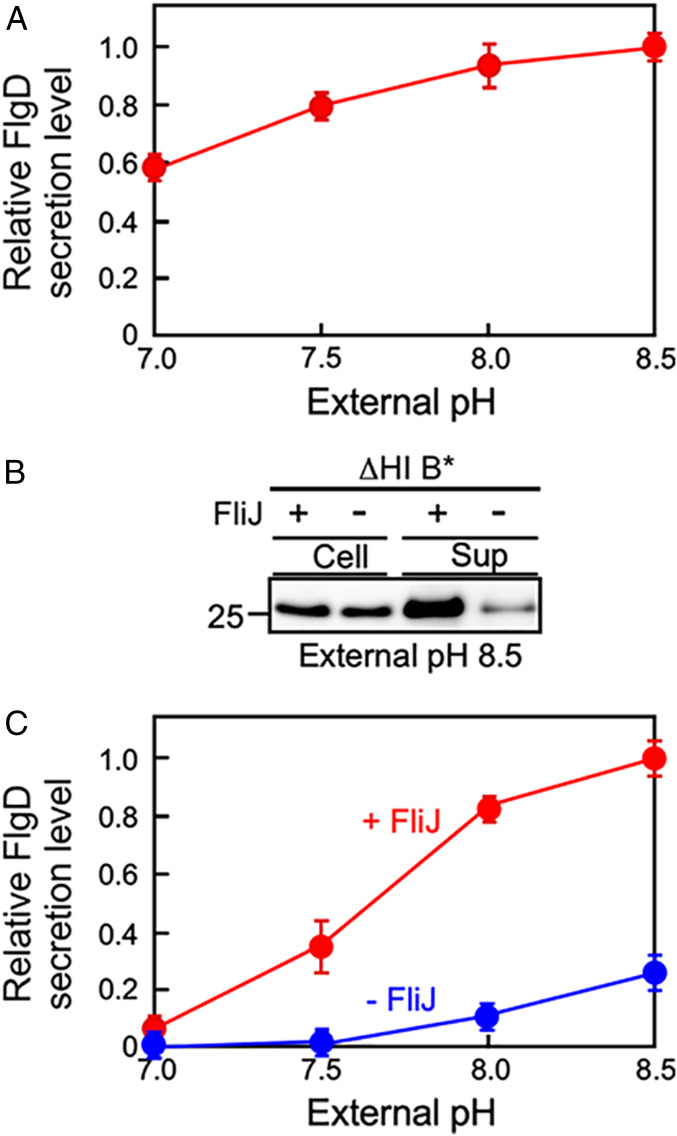
Identification of flagellar proteins involved in the Δψ-dependent activation mechanism of the transmembrane export gate complex. (*A*) The effect of FliJ overexpression on flagellar protein export by ∆HI B* cells. The HI B* cells overexpressing FliJ were grown at 30 °C in T-broth at external pH values of 7.0, 7.5, 8.0, or 8.5. The whole-cell and culture-supernatant fractions were analyzed by immunoblotting with polyclonal anti-FlgD antibody, and the relative secretion levels of FlgD were calculated. These data are the average from three independent experiments. The vertical bars indicate SDs. (*B* and *C*) Effect of the deletion of *fliJ* on flagellar protein export by ∆HI B* cells. The ∆HI B*(+ FliJ) and ∆HIJ B* (− FliJ) cells were grown at 30 °C in T-broth at external pH values of 7.0, 7.5, 8.0, or 8.5. The whole-cell and culture-supernatant fractions were analyzed by immunoblotting with polyclonal anti-FlgD antibody, and the relative secretion levels of FlgD were calculated. These data are the average from three independent experiments. The red line is taken from Fig. 1*A*.

An interaction between FliJ and FlhA_C_, which normally depends on FliH and FliI, turns the transmembrane export gate complex into a highly efficient Δψ-driven export engine (13). Therefore, we investigated whether overexpression of FliJ affects the Δψ dependence of flagellar protein export by the ΔHI B* mutant. Overexpression of FliJ significantly increased the secretion level of FlgD at an external pH of 7.0 to about 60% of the maximum level. An increase in the external pH from 7.0 to 8.5 further increased the FlgD secretion level by about 1.7-fold relative to the level at pH 7.0 (Fig. 3*A* and *SI Appendix*, Fig. S3). Deletion of *fliJ* from the ΔHI B* mutant decreased the maximum Δψ-dependent protein transport activity by about fourfold (Fig. 3*B*), but an increase in the external pH from 7.0 to 8.5 still caused an increase in the FlgD secretion level (Fig. 3*C* and *SI Appendix*, Fig. S2*D*). These results give further support to the idea that the transmembrane export gate is a voltage-gated protein transporter and demonstrate that FliJ is required for the membrane voltage-dependent activation mechanism of the export gate complex.

### Effect of a Gain-of-Function Mutation in FlhA on Δψ-Dependent Flagellar Protein Export by the ΔHI B* Mutant.

**Fig. 4.**
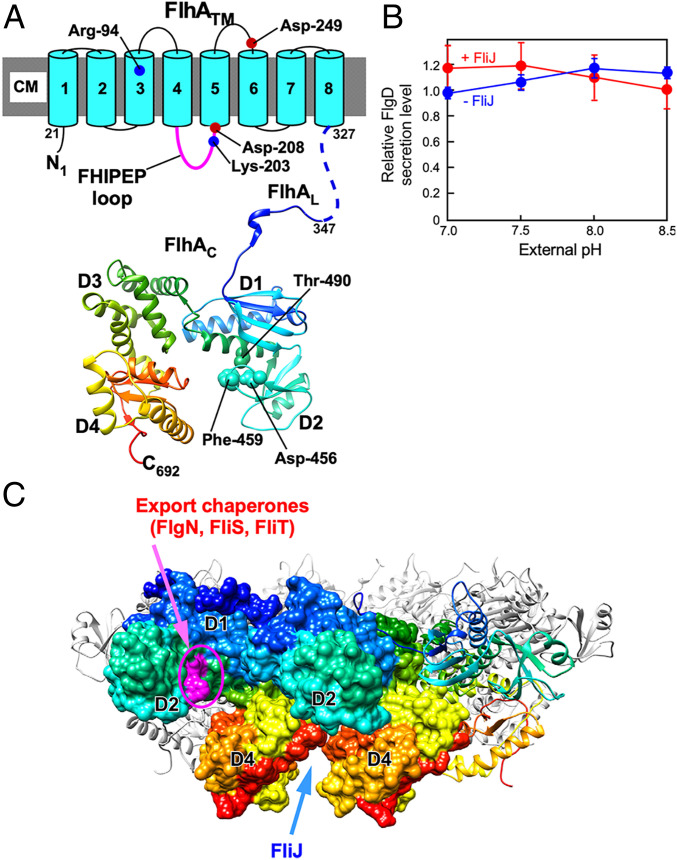
The effect of a gain-of-function mutation in FlhA on Δψ-dependent flagellar protein export. (*A*) The structure of FlhA. FlhA is composed of an N-terminal transmembrane region (FlhA_TM_) and a large C-terminal cytoplasmic domain (FlhA_C_). FlhA_C_ forms a docking platform for FliH, FliI, FliJ, export chaperones, and export substrates. FlhA_C_ (PDB ID 3A5I) consists of four domains, D1, D2, D3, and D4, and a flexible linker (FlhA_L_). The Cα backbone is color-coded from blue to red, going through the rainbow colors from the N terminus to the C terminus. The well-conserved Asp-456, Phe-459, and Thr-490 residues of FlhA are responsible for the interaction of FlhA_C_ with flagellar export chaperones in complex with their cognate substrates. The highly conserved Arg-94, Lys-203, Asp-208, and Asp-249 residues are critical for H^+^-coupled flagellar protein export. A highly conserved FHIPEP loop between transmembrane helices 4 and 5 of FlhA_TM_ binds to FlhA_C_ to coordinate the flux of H^+^ to flagellar protein export. (*B*) The effect of the *flhA(T490M)* mutation (A*) on Δψ-dependent flagellar protein export. The ∆HI B* A* (+ FliJ) and ∆HIJ B* A* (− FliJ) cells were grown at 30 °C in T-broth at external pH values of 7.0, 7.5, 8.0, or 8.5. The whole-cell and culture-supernatant fractions were analyzed by immunoblotting with polyclonal anti-FlgD antibody, and the relative secretion levels of FlgD were calculated. These data are the average from three independent experiments. (*C*) Model of the FlhA_C_ ring. The binding sites for flagellar export chaperones in complex with their cognate export substrates are shown in magenta. FliJ binds to a cleft between D4 domains of neighboring FlhA_C_ subunits.

FliJ binds to FlhA_C_ to facilitate H^+^-coupled protein export by the transmembrane export gate complex (13, 25). A gain-of-function mutation, *flhA(T490M)*, located in FlhA_C_ (Fig. 4*A*), overcomes the FliJ defect to a considerable degree (18, 26). Therefore, we investigated whether this gain-of-function mutation affects the Δψ dependence of flagellar protein export by the ΔHI B* mutant. To do so, we used the ΔHI B* cells containing the *flhA(T490M)* mutation (ΔHI B* A*). This mutant secreted a significant amount of FlgD into the culture supernatant even at an external pH of 7.0 (Fig. 4*B* and *SI Appendix*, Fig. S4*A*). The amount of FlgD secreted remained almost constant in the ΔHI B* A* mutant (Fig. 4*B*). As expected, the *fliJ* deletion did not significantly affect the secretion levels of FlgD by the ΔHI B* A* mutant (Fig. 4*B* and *SI Appendix*, Fig. S4*B*). These results suggest that the *flhA(T490M)* mutation allows FlhA to adopt a conformation mimicking the FliJ-bound state of FlhA, thereby eliminating the Δψ dependency of flagellar protein export by the export gate complex.

### Effect of *flhA(T490M)* on the H^+^ and Na^+^ Channel Activities of FlhA.

We found that the ΔHI B* A* mutant secreted a significant amount of FlgD into the culture media at an external pH of 7.0 (Fig. 4*B* and *SI Appendix*, Fig. S4*A*), raising the possibility that the *flhA(T490M)* mutation might affect the H^+^ and Na^+^ channel activities of FlhA. Because it has been reported that freely diffusing FlhA molecules in the cell membrane conduct both H^+^ and Na^+^ (17), we overexpressed wild-type FlhA and FlhA(T490M) in *Escherichia coli* BL21 (DE3) cells and measured intracellular pH and intracellular Na^+^ concentration using a ratiometric pH indicator probe, pHluorin(M153R) (27, 28), and a fluorescent Na^+^ indicator dye, CoroNa Green (17), respectively. The intracellular pH of the FlhA-overexpressing cells was 7.10 ± 0.06 (mean ± SD), which was ∼0.06 pH units lower than the internal pH of the vector control (7.16 ± 0.06) (*SI Appendix*, Fig. S5*A* and Table S3). This small pH drop was a statistically significant value (*P* = 0.037), indicating that free FlhA has H^+^ channel activity. The intracellular pH of the cells expressing FlhA with the *flhA(T490M)* mutation was essentially the same as that of the cells expressing wild-type FlhA (*SI Appendix*, Fig. S5*A*), indicating that this *flhA* mutation does not increase the H^+^ channel activity of FlhA.

Overexpression of wild-type FlhA caused a significant increment in the intracellular Na^+^ concentration in the presence of 100 mM NaCl but not in its absence (*SI Appendix*, Fig. S5*B* and Table S3), in agreement with a previous report (17). The intracellular Na^+^ concentration of the FlhA-overexpressing cells increased from 4.5 ± 2.1 mM (average ± SE; *n* = 30) to 73.1 ± 10.8 mM (*n* = 30) (*SI Appendix*, Fig. S5*B* and Table S3). The intracellular Na^+^ concentration of cells overexpressing FlhA with the *flhA(T490M)* mutation reached 75.6 ± 13.7 mM (*n* = 30) (*SI Appendix*, Fig. S5*B* and Table S3), again indicating that this residue change does not increase the intrinsic Na^+^ channel activity of FlhA. Consistently, the ΔHI B* A* mutant still showed a clear Na^+^ dependence for flagellar protein export at an external pH of 7.5 like the ΔHI B* mutant (*SI Appendix*, Fig. S6). Therefore, we propose that Δψ above the threshold value may act on FlhA_C_ to stabilize its interaction with FliJ to efficiently open the FlhA ion channel to conduct protons to drive flagellar protein export in a Δψ-dependent manner.

### Effect of the SMF on Flagellar Protein Export by the ΔHI B* Mutant.

Flagellar protein export by the ΔHI B* mutant shows a clear dependence on the external Na^+^ concentration over an external pH range of 6.0–8.0 (17, 18). Consistently, the secretion level of FlgD increased considerably when 100 mM NaCl was present in the medium (*SI Appendix*, Fig. S6). Neither Δψ nor total PMF changed upon addition of 100 mM NaCl (*SI Appendix*, Table S4). To analyze the impact of the SMF on the entire flagellar protein export process, we analyzed the number and length of the flagellar filaments produced by the ΔHI B* cells in the presence of 100 mM NaCl (Fig. 2 and *SI Appendix*, Table S2). The total SMF across the cell membrane was about 45 mV greater at an external pH of 8.5 than that at an external pH of 7.5 (*SI Appendix*, Fig. S7 and Table S4). About 73.5% of the Δ HI B* cells produced flagellar filaments, with an average of 1.4 ± 0.6 filaments per cell (mean ± SD; *n* = 125) at external pH 7.5 (Fig. 2*B*). The average filament length was 7.6 ± 2.5 μm (*n* = 50) (Fig. 2*C*). About 96.3% of the Δ HI B* cells produced filaments at an external pH of 8.5, with an average of 1.8 ± 0.8 per cell (*n* = 180) (Fig. 2*B*). The average filament length also increased significantly (9.0 ± 2.6 μm [*n* = 50]) at pH 8.5 (Fig. 2*C*). Thus, an increased Δψ also facilitates Na^+^-coupled protein export. At pH 8.5, the average filament length was significantly longer in the presence of 100 mM NaCl than in its absence (Fig. 2*C*). Because the SMF was larger than the PMF under our experimental conditions (*SI Appendix*, Table S4), we propose that the increased Δψ acts on the FlhA ion channel to facilitate the inward-directed flow of both H^+^ and Na^+^ when they are coupled to outward-directed protein translocation.

## Discussion

The Δψ component of the IMF is essential to supply energy for the translocation of ions, salts, proteins, and other molecules across the cell membrane. In animal cells, it is important for cell-to-cell communication as an electric signal transmitted to neighboring cells in cellular networks. In nerves, an increase in the membrane voltage above a threshold generates an action potential that is transmitted along the nerve axon. The action potential is generated by voltage-gated activation of ion channels. Interestingly, *Bacillus subtilis* utilizes a potassium channel to generate active, long-range electrical signaling in biofilms (23). This is the only biological function in bacteria observed to date in which a voltage-gated activation mechanism is involved. In the present study, we discovered that the flagellar protein export channel has a voltage-gated activation mechanism.

The flagellar protein export channel is intrinsically a dual-fuel engine that utilizes both H^+^ and Na^+^ as the coupling ion to drive flagellar protein export, and FlhA acts as a transmembrane ion channel to conduct both H^+^ and Na^+^ (17, 18). In the wild-type protein export apparatus, neither the ΔpH nor ΔpNa component is essential; the Δψ component is sufficient for flagellar protein export (13, 19). However, because ΔpH and ΔpNa become essential in the absence of the cytoplasmic ATPase complex (13, 17, 18), ΔpH and ΔpNa are thought to be required for efficient transit of H^+^ and Na^+^ across the cell membrane, respectively. However, it remained unknown when and how the export gate utilizes Δψ for ion-coupled protein export. Here, we used a *Salmonella* ΔHI B* mutant to study the role of Δψ in flagellar protein export. At an external pH of 7.0, the protein transport activity of the export gate is quite low in this mutant. An increase in the external pH from 7.0 to 8.5 increased Δψ by 1.5-fold (Fig. 1). When Δψ rose above a certain threshold, the export gate complex became an active protein export channel to drive H^+^-coupled protein export (Fig. 1). This result suggests that the flagellar protein export channel has a voltage-gated mechanism to activate H^+^-coupled protein export.

Overexpression of FliJ increased the secretion level of the hook-capping protein FlgD by the ΔHI B* mutant (Fig. 3*A*), whereas deletion of *fliJ* considerably decreased the secretion of FlgD over an external pH range of 7.5–8.5 (Fig. 3 *B* and *C*). Furthermore, the *flhA(T490M)* mutation in FlhA_C_ allowed the mutant to transport FlgD to a considerable degree even at an external pH of 7.0 (Fig. 4*B*). Deletion of *fliJ* in the ΔHI B* A* mutant did not affect the secretion level of FlgD over an external pH range of 7.0–8.5 (Fig. 4*B*). These results suggest that this *flhA* mutation may mimic the FliJ-bound state of FlhA that allows the export gate complex to be an active Δψ-driven protein export channel. Because FliJ binds to FlhA_C_ to facilitate H^+^-coupled protein export by the protein export channel complex (13, 25), we propose that an increase in Δψ acts on FlhA to stabilize the interaction between FlhA_C_ and FliJ, thereby opening the FlhA ion channel to facilitate the H^+^ flow that is coupled to flagellar protein export.

FlhA_C_ consists of the four domains, D1, D2, D3, and D4, and a flexible linker (FlhA_L_) that connects FlhA_C_ and FlhA_TM_ (Fig. 4*A*) (29). FlhA_C_ forms a homononameric ring in the flagellar protein export apparatus (Fig. 4*C*), and the FlhA_C_ ring projects into the central cavity of the basal body C-ring (*SI Appendix*, Fig. S1) (30, 31). Because the FlhA_C_ ring visualized in the flagellar basal body is far from the cytoplasmic membrane (32), the question arises how increased Δψ increases the binding affinity of FlhA_C_ for FliJ. A highly conserved sequence, called the FHIPEP loop, is located between transmembrane helices 4 and 5 of FlhA (Fig. 4*A*). The FHIPEP loop functionally communicates with FlhA_C_ during H^+^-coupled protein export (33), and so FlhA_C_ must get close to the cytoplasmic membrane through an interaction between FlhA_C_ and the FHIPEP loop. The crystal structure of a FliJ homolog, CdsO, in complex with CdsV_C_, which is a FlhA_C_ homolog, has led to the prediction that FliJ binds to a cleft between the D4 domains of neighboring FlhA_C_ subunits (Fig. 4*C*) (34). Thus, we propose that a larger Δψ may stabilize the interaction between FlhA_C_ and the FHIPEP loop, thereby allowing FliJ to bind to FlhA_C_ efficiently.

The highly conserved Arg-94, Asp-208, and Asp-249 residues of FlhA_TM_ are critical for H^+^-coupled protein export (35). The *flhA(K203A)* and *flhA(D208E*) mutations in the FHIPEP loop reduce the protein transport activity of the export gate complex, thereby slowing down protein export (35). The *flhA(D208A)* mutation increases the H^+^ channel activity of FlhA freely diffusing in the membrane (17), suggesting that the FHIPEP loop may control H^+^ translocation through the FlhA ion channel. Therefore, we propose that the FliJ–FlhA_C_ interaction induces conformational rearrangements of the FHIPEP loop in a Δψ-dependent manner, allowing FlhA_TM_ to become an active proton channel to drive flagellar protein export.

Flagellar export chaperones facilitate the docking of their cognate export substrates to FlhA_C_, as does the cytoplasmic ATPase complex consisting of FliH and FliI (36). The chaperone-binding site of FlhA_C_, which includes Asp-456, Phe-459, and Thr-490, is located at an interface between domains D1 and D2 (Fig. 4*A*) (37–39). Here, we showed that the *flhA(T490M)* mutation reduced the Δψ dependence of flagellar protein export by the ΔHI B* cells in a similar way as FliJ overexpression, suggesting that Thr-490 of FlhA attenuates the binding affinity of FlhA_C_ for FliJ, thereby creating the need for the Δψ-dependent activation of the FlhA ion channel. In the ΔHI B* mutant, the *flhA(T490M)* mutation considerably enhances the docking of proteins required for hook and basal body assembly to the FlhA_C_-FlhB_C_ docking platform of the export gate complex (26). This mutation thereby increases the probability of substrate entry into the polypeptide channel of the export gate complex. Therefore, we propose that the FliJ–FlhA_C_ interaction efficiently couples H^+^ flow through the FlhA_TM_ ion channel with substrate entry into the polypeptide channel.

## Materials and Methods

### Bacterial Strains, Plasmids, Transductional Crosses, and Media.

*Salmonella* strains and plasmids used in this study are listed in *SI Appendix*, Table S5. P22-mediated transductional crosses were carried out with p22HT*int*. T-broth contained 1% Bacto tryptone, 10 mM potassium phosphate. The pH of T-broth was adjusted to the desired final pH by addition of KOH.

### Secretion Assay.

A 50-μL volume of the overnight culture was inoculated into a 5-mL volume of fresh T-broth at an external pH value of 7.0, 7.5, 8.0, or 8.5 and incubated at 30 °C with shaking until the cell density had reached an OD_600_ of ∼1.4–1.6. Cultures were centrifuged to obtain cell pellets and culture supernatants. Cell pellets were resuspended in an SDS-loading buffer (62.5 mM Tris⋅HCl, pH 6.8, 2% SDS, 10% glycerol, 0.001% bromophenol blue) containing 1 μL of 2-mercaptoethanol, normalized to a cell density to give a constant number of cells. Proteins in the culture supernatants were precipitated by 10% trichloroacetic acid, suspended in a Tris/SDS loading buffer (1 vol of 1 M Tris, 9 vol of 1× SDS loading buffer) containing 1 μL of 2-mercaptoethanol and heated at 95 °C for 3 min. After sodium dodecyl sulfate–polyacrylamide gel electrophoresis (SDS-PAGE), immunoblotting with polyclonal anti-FlgD antibody was carried out using iBand Flex Western Device (Thermo Fisher Scientific). Detection was performed with Amersham ECL Prime Western blotting detection reagent (Cytiva). Chemiluminescence signals were captured by a Luminoimage analyzer LAS-3000 (GE Healthcare). The band intensity of each blot was analyzed using an image analysis software, CS Analyzer 4 (ATTO). More than three independent experiments were performed.

### Observation of Flagellar Filaments with a Fluorescent Dye.

The flagellar filaments produced by *Salmonella* cells were labeled using anti-FliC antiserum and anti-rabbit IgG conjugated with Alexa Fluor 594 (Invitrogen) as described previously (14). The cells were observed by fluorescence microscopy as described previously (40). Fluorescence images were analyzed using ImageJ software, version 1.53 (National Institutes of Health).

### Measurements of Δψ, Intracellular pH, and Intracellular Sodium Ion Concentration.

The Δψ component was measured using tetramethylrhodamine methyl ester (Invitrogen) as described previously (13). Intracellular pH measurements with a ratiometric fluorescent pH indicator protein, pHluorin(M153R), were carried out as described before (28). Intracellular sodium ion concentration was measured using CoroNa Green (Invitrogen) as described previously (41).

### Statistical Analysis.

Statistical analyses were done using Prism 7.0c software (GraphPad). Comparisons were performed using a two-tailed Student *t* test. A value of *P* < 0.05 was considered to be a statistically significant difference. *P < 0.05; **P < 0.01; ***P < 0.001.

## Supplementary Material

Supplementary File

## Data Availability

All study data are included in the article and/or supporting information.
