# Supplementary material for: Membrane voltage-dependent activation mechanism of the bacterial flagellar protein export apparatus
Source: Proc Natl Acad Sci U S A. 2021 May 25;118(22):e2026587118. doi: 10.1073/pnas.2026587118 (PMC8179193; doi:10.1073/pnas.2026587118)
Supplement: Supplementary File [file pnas.2026587118.sapp.pdf]

Supplementary Information

Membrane voltage-dependent activation mechanism of the bacterial flagellar protein export apparatus

Tohru Minamino, Yusuke V. Morimoto,

Miki Kinoshita and Keiichi Namba

Corresponding author: T. Minamino, K. Namba.

E-mail: tohru@fbs.osaka-u.ac.jp (to T.M.); keiichi@fbs.osaka-u.ac.jp (K.N.).

This PDF file includes:

Figures S1 to S7

Tables S1 to S5

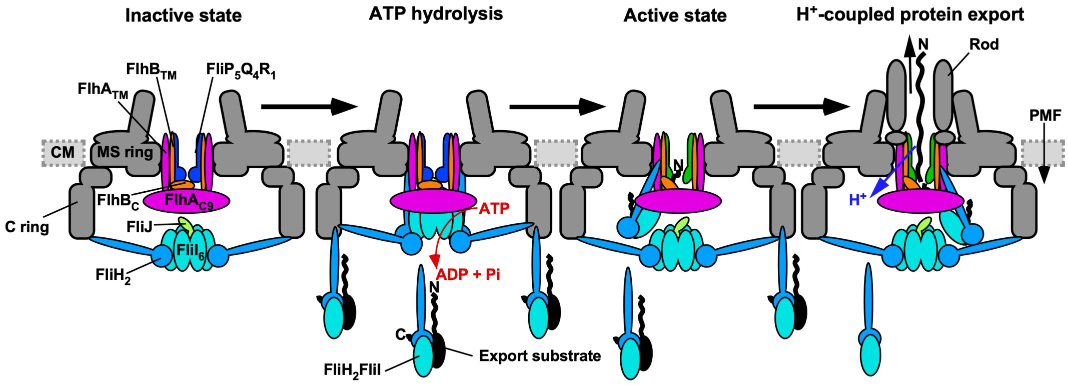

Fig. S1. Energy-coupling mechanism of the flagellar protein export apparatus. The transmembrane export gate complex is located inside the basal body MS ring and utilizes the proton motive force (PMF) across the cytoplasmic membrane (CM) to drive flagellar protein export. FliP, FliQ and FliR form the $FliP_5FliQ_4FliR_1$ complex. The N-terminal transmembrane domain of FlhB ($FlhB_{TM}$) associates with the $FliP_5FliQ_4FliR_1$ complex, and its C-terminal cytoplasmic domain ($FlhB_C$) projects into the central cavity of the C ring. FlhA forms a homo-nonamer through interactions between the C-terminal cytoplasmic domain of FlhA ($FlhA_C$), and its N-terminal transmembrane domain ($FlhA_{TM}$) forms a pathway for the transit of protons (H^+) from the periplasm to the cytoplasm. The cytoplasmic ATPase ring complex consisting of FliH, FliI and FliJ associates with the C-ring through an interaction between FliH and a C ring protein, FliN. When the flagellar protein export apparatus is inactive, both polypeptide and proton channels of the export gate complex are closed (left, step 1). ATP hydrolysis by the $FliI_6$ ring induces conformational rearrangements of the export gate complex through interactions between FliJ and $FlhA_C$ (middle left, step 2), allowing the gate complex to become an active proton/protein antiporter that couples the proton flow through the FlhA proton channel to the translocation of flagellar building blocks into the polypeptide channel of the $FliP_5FliQ_4FliR_1$ complex (middle right, step 3). The $FliH_2FliI$ complex freely diffuses in the cytoplasm and brings export substrates from the cytoplasm to the export gate complex through interactions of FliH with FliN, as well as with $FlhA_{TM}$. The functionally active export gate complex efficiently utilizes PMF to unfold and transport each building block into the central channel of the growing flagellar structure (right, step 4).

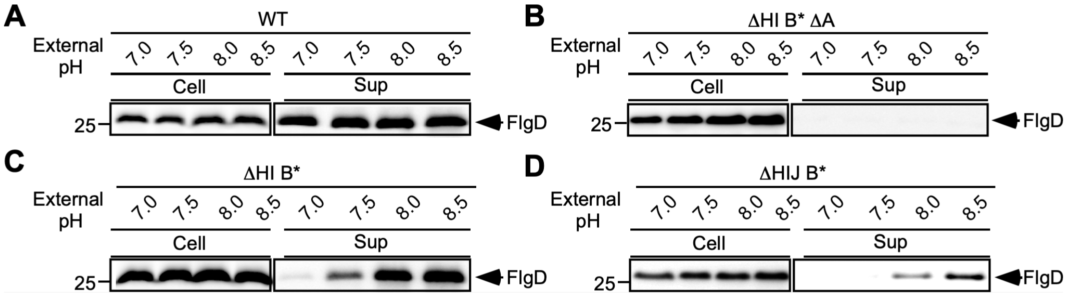

Fig. S2. Effect of Δψ on flagellar protein export. Immunoblotting, using polyclonal anti-FlgD antibody, of whole cell proteins (Cell) and culture supernatant fractions (Sup) prepared from (A) SJW1103 (WT), (B) NH004 (ΔHI B* ΔA), (C) MMHI0117 (ΔHI B*) and (D) MMHIJ0117 (ΔHIJ B*) grown exponentially at 30ºC in T-broth at an external pH value of 7.0, 7.5, 8.0 or 8.5 in the absence of 100 mM NaCl.

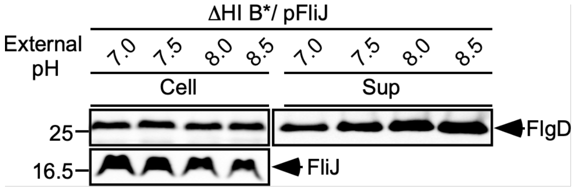

Fig. S3. Multicopy effect of FliJ on Δψ-dependent flagellar protein export by the ΔHI B* mutant. Immunoblotting, using polyclonal anti-FlgD or anti-FliJ antibody, of whole cell proteins (Cell) and culture supernatant fractions (Sup) prepared from MMHI0117 cells carrying pMM404 (ΔHI B*/ pFliJ) grown exponentially at 30ºC in T-broth at an external pH value of 7.0, 7.5, 8.0 or 8.5 in the absence of 100 mM NaCl.

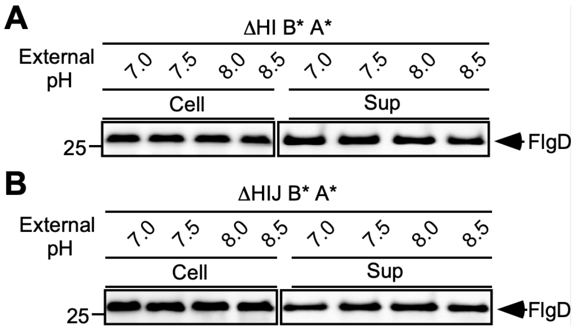

Fig. S4. Effect of the *flhA(T490M)* mutation (A*) on Δψ-dependent flagellar protein export. Immunoblotting, using polyclonal anti-FlgD antibody, of whole cell proteins (Cell) and culture supernatant fractions (Sup) prepared from (A) MMHI0117-3 (ΔHI B* A*), (B) MMHIJ0117-3 (ΔHIJ B* A*) grown exponentially at 30ºC in T-broth at an external pH value of 7.0, 7.5, 8.0 or 8.5 in the absence of 100 mM NaCl.

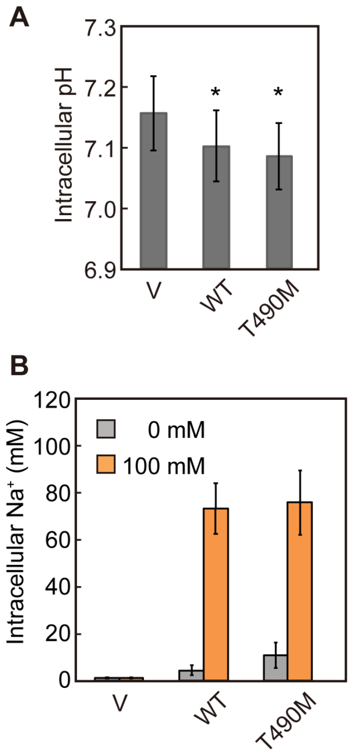

Fig. S5. Effect of gain-of-function mutations in FlhA on the H^+ and Na^+ channel activities of FlhA. (A) Effect of overexpression of FlhA on intracellular pH. Intracellular pH was measured with pHluorin at an external pH of 5.5. The BL21(DE3) strain expressing the pHluorin probe was transformed with pBAD24 (Vector, V), pNH319 (FlhA, indicated as WT) or pNH319(T490M) [FlhA(T490M), indicated as T490M]. Vertical bars indicate standard deviations of twelve independent biological replicates. The data that exhibited a statistically significant intracellular change compared with the vector control are highlighted with an asterisk ($P < 0.05$). (B) Effect of overexpression of FlhA on intracellular Na^+ concentration. The intracellular Na^+ concentration was measured with CoroNa Green in the presence and absence of 100 mM NaCl at external pH 7.0. The BL21(DE3) strain was transformed with the above plasmids. For each transformant, 30 cells were measured. Vertical bars indicate standard errors. (See SI Appendix, Table S3)

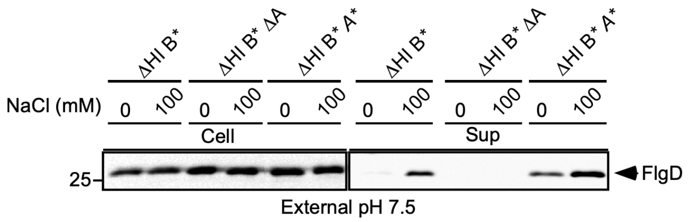

Fig. S6. Effect of the *flhA(T490M)* mutation (A*) on Na⁺-coupled flagellar protein export. Immunoblotting, using polyclonal anti-FlgD antibody, of whole cell proteins (Cell) and culture supernatant fractions (Sup) prepared from ΔHI B*, ΔHI B* ΔA, ΔHI B* A* grown exponentially at 30ºC in T-broth (pH 7.5) with or without 100 mM NaCl.

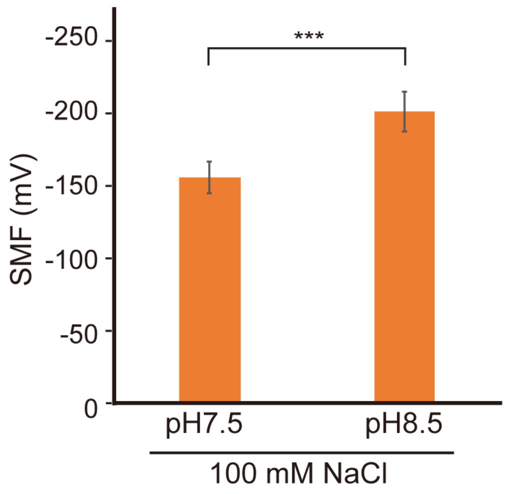

Fig. S7. Measurements of SMF. The intracellular Na^+ concentration was measured with CoroNa green. More than 100 ΔHI B* cells were measured. Vertical bars indicate standard deviations. Vertical bars indicate standard deviations. Comparisons between datasets were performed using a two-tailed Student's *t*-test. A *P* value of < 0.05 was considered to be a statistically significant difference. ***, *P* < 0.001. (See SI Appendix, Table S4)

Table S1. Membrane potential, intracellular pH and total PMF over an external pH value of 7.0–8.5.

Strain	pH_{ex}	7.0	7.5	8.0	8.5
WT	pH_{in}	7.40 ± 0.01 (n = 4)	7.45 ± 0.03 (n = 4)	7.47 ± 0.12 (n = 4)	7.50 ± 0.05 (n = 4)
	Membrane potential (mV)	-87.9 ± 10.5 (n = 936)	-99.4 ± 11.5 (n = 603)	-130.1 ± 12.8 (n = 328)	-135.2 ± 11.5 (n = 484)
	PMF (mV)	-111.1 ± 11.1	-96.6 ± 13.1	-98.9 ± 19.8	-76.4 ± 14.2
ΔHI B*	pH_{in}	7.39 ± 0.03 (n = 4)	7.45 ± 0.03 (n = 4)	7.49 ± 0.05 (n = 4)	7.55 ± 0.04 (n = 4)
	Membrane potential (mV)	-93.5 ± 9.8 (n = 1208)	-106.4 ± 11.2 (n = 770)	-127.0 ± 11.0 (n = 335)	-133.4 ± 12.2 (n = 737)
	PMF (mV)	-116.2 ± 11.3	-103.3 ± 13.1	-97.3 ± 13.8	-77.9 ± 14.7

Proton motive force is defined as equation [1].

$$PMF = V_m + \frac{k_B T}{q} \, ln \frac{[H^+]_{in}}{[H^+]_{ex}} \qquad [1]$$

where V_m is $\Delta\psi$, $[H^+]_{in}$ and $[H^+]_{ex}$ are the internal and external ion concentrations, respectively, k_B is the Boltzmann's constant, T is the absolute temperature (K) and q is the charge of ions.

Table S2. Effects of external pH and external Na^+ concentration on flagellar formation.

Strain	pH_{ex}	NaCl (mM)	Fraction of flagellated cells (%)	Average number of flagella in flagellated cell (mean ± SD)	Average length of filament (µm) (mean ± SD)
ΔHI B*	7.5	0	1.0 (n = 198)	1.0 ± 0.0 (n = 2)	-
		100	73.5 (n = 170)	1.4 ± 0.6 (n = 125)	7.6 ± 2.5 (n = 50)
	8.5	0	60.5 (n = 177)	1.3 ± 0.5 (n = 107)	4.8 ± 1.5 (n = 50)
		100	96.3 (n = 187)	1.8 ± 0.8 (n =1 80)	9.0 ± 2.6 (n = 50)

pH_{ex}, external pH

Table S3. Ion conductivity of FlhA mutants.

<table>
<tr><th colspan="2"></th><th>V</th><th>WT</th><th>T490M</th></tr>
<tr><td colspan="2">Internal pH
(mean ± SD)</td><td>7.16 ± 0.06
(n = 12)</td><td>7.10 ± 0.06
(n = 12)</td><td>7.09 ± 0.05
(n = 12)</td></tr>
<tr><td rowspan="2">$[Na^{+}]_{in}$ (mM)
(mean ± SEM)</td><td>NaCl,
0 mM</td><td>1.48 ± 0.03
(n = 30)</td><td>4.5 ± 2.1
(n=30)</td><td>10.9 ± 5.3
(n = 30)</td></tr>
<tr><td>NaCl,
100 mM</td><td>1.51 ± 0.03
(n = 30)</td><td>73.1 ± 10.8
(n = 30)</td><td>75.6 ± 13.7
(n = 30)</td></tr>
</table>

V, vector control.

Table S4. Ion motive force in *Salmonella* HI B* cells

Strain	pH_{ex}	7.5		8.5	
	$[Na^+]_{ex}$ (mM)	0	100	0	100
ΔHI B*	pH_{in}	7.43 ± 0.12 (n = 4)	7.43 ± 0.12 (n = 4)	7.54 ± 0.03 (n = 4)	7.54 ± 0.03 (n = 4)
	Membrane potential (mV)	-92.1 ± 5.6 (n = 71)	-91.6 ± 3.3 (n = 270)	-140.7 ± 5.0 (n = 201)	-137.7 ± 6.0 (n = 144)
	$[Na^+]_{in}$ (mM)	4.2 ± 0.4	8.0 ± 12.9	4.2 ± 0.4	8.0 ± 12.9
	PMF (mV)	-88.1 ± 10.5	-87.6 ± 10.5	-84.5 ± 4.8	-81.5 ± 7.5
	SMF (mV)	-	-155.8 ± 10.9	-	-201.9 ± 13.6

Ion motive force is defined as equation [2].

$$IMF = V_m + \frac{k_B T}{q}\, ln \frac{[ion]_{in}}{[ion]_{ex}} \qquad [2]$$

where V_m is $\Delta\psi$, $[ion]_{in}$ and $[ion]_{ex}$ are the internal and external ion concentrations, respectively, k_B is the Boltzmann's constant, T is the absolute temperature (K) and q is the charge of ions.

Table S5. Strains and plasmids used in this study

Strains/ Plasmids	Relevant characteristics	Source or reference
E. coli		
BL21 (DE3)	Over-expression of proteins	Novagen
Salmonella		
SJW1103	Wild type for motility and chemotaxis	(42)
MMHI0117	*ΔfliH-fliI flhB(P28T)*	(20)
MMHI0117-3	*ΔfliH-fliI flhB(P28T) flhA(T490M)*	(26)
MMHIJ0117	*ΔfliH-fliI-fliJ flhB(P28T)*	(13)
MMHIJ0117-3	*ΔfliH-fliI-fliJ flhB(P28T) flhA(T490M)*	(18)
NH004	*ΔfliH-fliI flhB(P28T) ΔflhA*	(35)
**Plasmids**		
pBAD24	Expression vector	(43)
pTrc99AFF4	Expression vector	(44)
pMM404	pTrc99AFF4/ FliJ	(45)
pNH319	pBAD24/ N-His-FLAG-FlhA	(17)
pNH319(T490M)	pBAD24/ N-His-FLAG-FlhA(T490M)	This study
pYC17	pACTrc/pHluorin	(17)
pYVM001	pKK223-3/pHluorin(M153R)	(28)

References

42. S. Yamaguchi, H. Fujita, K. Sugata, T. Taira, T. Iino, Genetic analysis of *H2*, the structural gene for phase-2 flagellin in *Salmonella*. *J. Gen. Microbiol.* **130**, 255–265 (1984).

43. L. M.Guzman, D. Belin, M. J. Carson, J. Beckwith, Tight regulation, modulation, and high-level expression by vectors containing the arabinose PBAD promoter. *J. Bacteriol.* **177**, 4121–4130 (1995).

44. K. Ohnishi, F. Fan, G.J. Schoenhals, M. Kihara, R.M. Macnab, The FliO, FliP, FliQ, and FliR proteins of *Salmonella typhimurium*: putative components for flagellar assembly. *J. Bacteriol.* **179**, 6092–6099 (1997).

45. T. Minamino, R. Chu, S. Yamaguchi, R.M. Macnab, Role of FliJ in flagellar protein export in *Salmonella*. *J. Bacteriol.* **182**, 4207-4215 **(**2000).
